# The neutrophil-to-lymphocyte ratio (NLR) predicts adrenocortical carcinoma and is correlated with the prognosis

**DOI:** 10.1186/s12894-017-0240-4

**Published:** 2017-06-29

**Authors:** Taku Mochizuki, Takashi Kawahara, Daiji Takamoto, Kazuhide Makiyama, Yusuke Hattori, Jun-ichi Teranishi, Yasuhide Miyoshi, Yasushi Yumura, Masahiro Yao, Hiroji Uemura

**Affiliations:** 1 Departments of Urology and Renal Transplantation, Yokohama City University Medical Center, 4-57 Urafune-cho, Minami-ku, Yokohama, Kanagawa 2320024 Japan; 2 Department of Urology, Yokohama City University, Graduate School of Medicine, Yokohama, Japan

**Keywords:** Biomarker, Neutrophil-to-lymphocyte ratio, Adrenal tumor, Adrenocortical carcinoma

## Abstract

**Background:**

The neutrophil-to-lymphocyte ratio (NLR) is reported as a biomarker for some solid malignant diseases. Thus far, however, no reports of the relationship between the NLR and adrenal tumors have been published. We analyzed the utility of the preoperative NLR as a biomarker for predicting the prognosis or diagnosis of malignant disease.

**Methods:**

A total of 59 patients with adrenal tumors (13 cases of malignant disease and 46 with benign disease) were analyzed in this study from February 2004 to June 2015 at our institute. The NLR was obtained just before adrenalectomy. The diagnosis of adrenal tumor was confirmed by a pathological examination of surgical specimens.

**Results:**

The NLR in malignant adrenal tumor specimens was significantly higher than in non-malignant specimens (*p* = 0.028). Adrenocortical carcinoma (ACC) showed the highest NLR among all adrenal tumors. In ACC, the higher NLR group (NLR ≥ 5) showed a significantly poorer overall survival than the lower NLR group (NLR < 5) (*p* = 0.032).

**Conclusions:**

In adrenal tumors, a higher NLR indicates a higher incidence of malignancy. The NLR might be a new biomarker for predicting the prognosis of adrenal tumor patients.

## Background

Adrenocortical carcinoma (ACC) is a very rare disease with an incidence of 0.5 to 2.0 patients/100,000,000 patients/year and has one of the poorest prognoses among all solid malignancies [1, 2]. A large number of patients are diagnosed at an advanced stage. About 70% of ACCs are reported to be diagnosed as extra-adrenal lesions [3]. However, despite this poor prognosis, some patients have achieved a long disease-free survival [4]. There were no confirmed risk to predict a poorer prognosis. Several candidate prognostic factors of ACC have been reported, including the tumor size, rate of Ki-67 positivity, completeness of resection of the surgical margin, and the clinical stage [5].

A number of studies have revealed that the neutrophil-to-lymphocyte ratio (NLR) is an independent prognostic factor in various solid malignancies, including prostatic carcinoma and renal cell carcinoma [6, 7]. The NLR has also been suggested to be not only a predictor of the systemic inflammatory response in critical care patients but also a prognostic factor for certain solid malignancies [8–10]. The NLR can be easily calculated from routine complete blood counts (CBCs) in peripheral blood.

The aims of this study were to compare the NLR in ACC patients with those in patients with other non-malignant adrenal tumors and to evaluate the NLR as a prognostic marker in ACC patients.

## Methods

### Patients

A total of 59 consecutive cases, including 33 adrenocortical adenoma, 13 pheochromocytoma, 9 adrenocortical cancer, and 4 malignant lymphoma, at our institute from February 2004 to June 2015 were analyzed in this study.

### Clinical and laboratory assessments

The NLR was calculated using the neutrophil and lymphocyte counts obtained via CBCs just before adrenalectomy as part of the routine preoperative workup of patients. We set the cut-off point of 5.0 as the threshold defining an elevated NLR, in accordance with a previous report [11]. None of the patients demonstrated systemic inflammation or blood disease at the time of the blood examinations.

### Statistical analyses

The patients’ characteristics were analyzed using the Mann-Whitney U and chi-squared tests. The cut-off NLR was determined based on the results of a receiver operative curve (ROC) analysis and a multivariate analysis was performed to investigate the factors associated with malignancy in adrenal tumors. Spearman’s correlation coefficients were calculated to investigate the correlation between the NLR and tumor size. The Kaplan-Meier product limit estimator was used to estimate the overall survival. A log-rank test was performed for comparison. The statistical analyses were performed using the Graph Pad Prism software program (Graph Pad Software, La Jolla, CA, USA). *P* values of <0.05 were considered to indicate statistical significance.

## Results

### Patients

We examined a total of 59 cases of adrenal tumor, including 33 cases of adrenal carcinoid (mean size, 22 mm; median (mean ± standard deviation) NLR, 2.84 (3.41 ± 1.89)), 13 cases of pheochromocytoma (mean size, 40 mm; NLR, 2.03 (2.47 ± 1.54)), nine cases of ACC (mean size, 100 mm; NLR, 6.02 (5.04 ± 3.09)), and four cases of malignant lymphoma (mean size, 91 mm; NLR, 3.30 (3.82 ± 2.18)) [Table 1]. Regarding the ACC cases, 8 (88.9%) were female and one (11.1%) was male. One of the four patients showed endocrine activity and capsule invasion. The median age was 64 years old, and the median follow-up period was 17 months. The ACC patients’ backgrounds, including the tumor volume and clinical stage, are shown in Table 2.

**Table 1:** Each adrenal tumor’s characteristics

	adrenocortical adenoma	pheochromocytoma	adrenocortical carcinoma	malignant lymphoma
n	33	13	9	4
Sex (male: female)	10: 23	6: 7	1: 8	3: 1
Age (median)	50	57	64	70
Maximum tumor diameter (mm) (mean)	22	40	100	91
NLR (median)	2.84	2.03	6.02	3.30

**Table 2:** Adrenocortical carcinoma patients’ characteristics

No	Age	Sex	Maximum diameter (mm)	Endocrine activity	Capsule invasion	Clinical stage	Chemotherapy	Outcome	Observation period (months)
1	85	F	96	−	NA	StageIV	−	dead	1
2	19	F	125	+	+	StageIII	+	dead	5
3	64	F	72	−	NA	StageIV	+	dead	42
4	62	M	100	−	+	StageIV	+	dead	9
5	64	F	105	−	NA	StageIV	+	dead	2
6	66	F	70	−	+	StageIV	+	dead	30
7	42	F	108	−	−	StageIV	+	dead	17
8	34	F	125	−	−	StageIV	−	dead	17
9	72	F	18	−	+	StageI	−	alive	27

### NLR values and patient outcomes

**Fig. 1:**
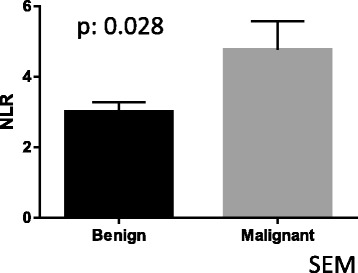
Comparison NLR of malignant adrenal tumor to NLR of non-malignant adrenal tumor

**Fig. 2:**
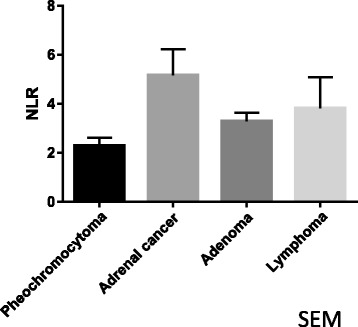
Comparison NLR of adrenocortical carcinoma, pheochromocytoma, adrenal malignant lymphoma and adrenal adenoma

**Fig. 3:**
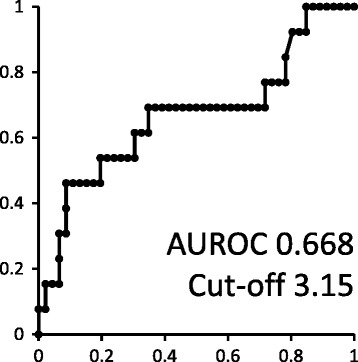
Receiver operator characteristics curve to detect cut off point of NLR to detect malignant disease or not (area under ROC: 0.668)

**Fig. 4:**
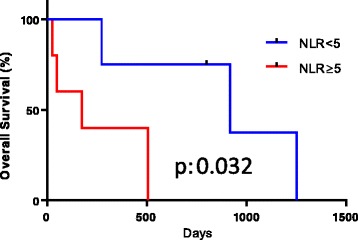
Overall survival of adrenocortical carcinoma according to the NLR

Malignant adrenal tumors showed a significantly higher NLR (mean ± standard deviation, standard error of the mean: 4.76 ± 2.93, 0.81) than non-malignant adrenal tumors (3.01 ± 1.78, 0.27; *p* = 0.016). The NLR in ACC showed the highest NLR [Figs. 1 and 2]. The cut-off NLR for predicting malignant disease was 3.15 (area under ROC: 0.668) [Fig.3]. A multivariate analysis showed that the NLR was an independent predictor (GR 8.990, *p*: 0.037) (Table 3). The tumor size was weakly correlated with the NLR (r^2^: 0.08, *p*: 0.034). In ACC subjects, the higher NLR group (≥ 5) showed a significantly poorer overall survival than the lower NLR group (< 5) with a median survival of 174 vs. 917 days (*p* = 0.032) [Fig. 4].

**Table 3:** The results of the multivariate analysis

	*P* value	HR	95% CI
Gender (male)	0.332	0.353	0.043–2.892
Age (≥57)	0.099	5.720	0.719–45.504
NLR (≥3.15)	0.037	8.990	1.142–70.547
Size (≥40 mm)	<0.001	112.400	7.39–1710.5

## Discussion

Recently, NLR had been shown to be an independent prognostic risk factor for certain solid malignancies [6–10]. Only one study comparing the NLR and the prognosis of ACC has been reported—by Bagante et al. [11]. They found that an NLR > 5.0 was associated with a poorer disease-specific survival and progression-free survival in ACC. No other reports have investigated the effectiveness of NLR in detecting the malignancy of an adrenal tumor. In the present study, we found that a higher NLR in adrenal tumors was associated with a higher incidence of malignancy. These findings might contribute to the prediction of malignant disease for differentiating incidentaloma.

Among incidental adrenal tumors, ACC is difficult to diagnose using preoperative imaging findings. A tumor size >4 cm is a well-known imaging finding for differentiating malignant tumors, with a sensitivity of 81% [5]. A previous report showed that 16% of adrenal tumors with diameters of <5 cm were ACCs [12].

Due to the marked increase in the rate of imaging analyses being performed at medical check-ups, small ACC would be increased. So, differentiated diagnosis except tumor size would be needed.

Irregular margins are usually seen in ACC, but some benign adrenal tumors also showed irregular margins [13]. Computed tomography (CT) shows a finding of a low Hounsfield unit value (< 10) with 98% specificity. Chemical-shift imaging with magnetic resonance imaging (MRI) has also been reported to be useful for detecting adrenal adenomas [14]. However, the specific imaging findings indicative of ACC remain unclear. The NLR can be easily calculated during a daily clinical examination. The combination of imaging findings on CT and/or MRI and the NLR may support the preoperative diagnosis of adrenal tumor.

Previous reports have shown that an NLR > 5.0 indicates a poor prognosis in pancreas cancer and liver metastatic rectal cancer [15, 16]. Our previous study showed that an NLR > 2.4 was associated with a high risk of prostate cancer in patients with a PSA of 4–10 ng/mL [17]. However, due to the small number of patients, that study could not detect an adequate NLR cut-off point. In the present study, we found that an NLR cut-off point of 5.0 was adequate for predicting the prognosis of ACC. The cut-off NLR of 5.0 was the same as a previous report and was the median value in this study. The cut-off NLR of 5.0 was relatively high in comparison to other studies, which indicated the aggressive nature of ACC in comparison to other types of cancer. Further studies are needed to validate the clinical utility of this parameter.

In this study, the multivariate analysis revealed that the tumor size and NLR were found to be independent predictors of malignant disease. The combination of the NLR and imaging findings might support the preoperative diagnosis and detection of malignant adrenal tumors, including ACC and malignant lymphoma, as well as benign adrenal tumors, helping in the planning of an adequate surgical approach.

This study showed that an NLR of 5.0 was a candidate cut-off point for predicting the prognosis in ACC. In localized ACC, the surgical margin was confirmed to be the most important prognostic factor. The 5-year overall survival in completely surgically resected patients ranges from 40 to 50%, while the median overall survival in unresectable case is <1 year [6]. In cases preoperatively predicted to have a poor outcome, a preoperative surgical plan with extended resection might be suggested in order to obtain a negative surgical margin.

This study was limited by its small sample size and retrospective nature, both due to the rarity of ACC. Further study is needed to confirm the usefulness of NLR in ACC to confirm the sensitivity and specificity.

## Conclusion

Malignant adrenal tumors showed a higher NLR than non-malignant ones. In addition, the ACC patients with a higher NLR showed a significantly poorer survival than those with lower values.
